# Cookies, Chips, and Seeds: How Human Food Leftovers Influence Ant-Mediated Seed Removal

**DOI:** 10.3390/biology15080657

**Published:** 2026-04-21

**Authors:** Brenda Morris, Damaris Iturralde, Anabel Almanza, Aslithe Henriquez, María Morales, Digna Rodríguez, Héctor Santos, Joseph Yángüez, Ronny Castillo, Carlos A. Gómez, Pedro González, Cristie Rodríguez, Solmaira Acosta, Adolfo Alba, Lara Dominguez, Emily Marple, Dumas Gálvez

**Affiliations:** 1 Programa Centroamericano de Maestría en Entomología (PCMENT), Vicerrectoría de Investigación y Postgrado, Universidad de Panamá, Panama City 0819-07289, Panama; brenda4mn@gmail.com (B.M.); damarisestefania23@gmail.com (D.I.); anabelalmanza28@gmail.com (A.A.); aslithehenriquez@gmail.com (A.H.); mariiaisa14@gmail.com (M.M.); dignamr09@gmail.com (D.R.); hectorsantos0004@gmail.com (H.S.); josephadrian99@outlook.es (J.Y.); ronnyalbertoc@gmail.com (R.C.); carlosariel031211@gmail.com (C.A.G.); pedroar1400@gmail.com (P.G.); crimili46@gmail.com (C.R.); solserrano325@gmail.com (S.A.); nombre9820@gmail.com (A.A.); 2 Smithsonian Tropical Research Institute, Balboa, Ancón, Panama City 0843-03092, Panama; lara.blair.dominguez@gmail.com (L.D.); emilymeymarple@gmail.com (E.M.); 3 Coiba Scientific Station, City of Knowledge, Calle Gustavo Lara, Building 145B, Clayton 0843-01853, Panama

**Keywords:** ant-plant interaction, food subsidies, myrmecochory, urban ecology, seed dispersal

## Abstract

Ants play a central role in seed dispersal and predation, shaping plant recruitment, yet their foraging behavior is increasingly influenced by anthropogenic food subsidies. In human-modified landscapes, processed food waste may disrupt ant–seed interactions by diverting foragers or altering activity patterns, but the extent and mechanisms of these effects across habitats remain unclear. We conducted three field experiments in Panama to test how common food residues affect seed removal by ants in urban and forest environments. Using oat seeds as standardized diaspores, we (1) tested whether potato chips surrounding seed depots reduced removal, (2) evaluated the effects of adjacent chips or cookies on removal rates, ant activity, and species composition, and (3) manipulated the distance between chips and seeds (0, 30, 60 cm) to distinguish behavioral distraction from physical obstruction. Across experiments, seeds near food residues were removed significantly more slowly than controls, approximately half as fast in both habitats, despite differences in ant assemblages. Ant activity near seeds declined in the presence of food, particularly in the urban site. Suppression of seed removal occurred at close range but disappeared at 60 cm. These findings indicate that food waste disrupts ant-mediated seed removal through fine-scale behavioral shifts across contrasting habitats.

## Simple Summary

Ants play an important role in forests and cities because they move and bury seeds, helping many plants grow and regenerate. However, in places where people leave behind processed foods such as cookies and potato chips, ants may change their behavior. We wanted to understand whether these common food leftovers affect how ants handle seeds. To test this, we carried out field experiments in both a tropical forest and an urban area in Panama. We placed seeds on the ground either alone or near cookies or chips and measured how quickly ants removed them. We also tested whether the distance between seeds and food changed the outcome. We found that when human food was nearby, ants removed seeds much more slowly, in both forest and city sites. This effect was strongest when the food was placed right next to the seeds. Our results show that everyday food waste can interfere with natural interactions between ants and plants. Reducing food litter in natural and urban areas may therefore help maintain healthy ecosystems and plant regeneration.

## 1. Introduction

Ants are among the most abundant and ecologically influential organisms in terrestrial ecosystems, where they play central roles in nutrient cycling, soil turnover, and plant community dynamics [1]. One of their most important contributions to plant ecology is seed removal, which can result in either seed dispersal (myrmecochory) or seed predation (granivory), depending on ant species, seed traits, and environmental context. Through these interactions, ants strongly influence seed fate, spatial redistribution, and ultimately plant recruitment and community composition, making ant foraging behavior a critical but vulnerable component of plant regeneration processes [2].

In myrmecochorous systems, ants and flowering plants have evolved a tight mutualism in which seeds bear lipid-rich appendages (elaiosomes) that attract ants. Ants transport seeds to their nests, consume the elaiosome, and discard the intact seed in nutrient-rich, enemy-reduced microsites, thereby enhancing seed survival and establishment [3]. However, ants also remove and transport seeds that lack elaiosomes, acting as secondary dispersers or predators across a wide range of ecosystems [4]. In these non-myrmecochorous interactions, seed removal is shaped by ant foraging selectivity, which depends on diaspore size, mass, handling costs, and nutritional content [5,6,7].

Ant foraging behavior, and thus seed removal, is highly plastic and responsive to changes in resource availability. In urban ecosystems, this plasticity is increasingly shaped by the widespread availability of anthropogenic food subsidies, including processed food waste rich in carbohydrates and lipids [8,9]. Stable isotope analyses have shown that urban ants incorporate substantial amounts of human-derived resources into their diets, often exhibiting isotopic signatures consistent with C4 crops commonly used in processed foods [8]. Experimental work in urban parks and pavements has further demonstrated that ants rapidly exploit foods such as potato chips and cookies, removing them at high rates comparable to or exceeding those of natural resources [9].

The ecological consequences of this dietary shift remain poorly understood, particularly for ant-mediated interactions involving plants. Because ants are key agents of both seed dispersal and seed predation, the presence of highly attractive, calorie-dense food waste may alter their foraging priorities and weaken ant–seed interactions. Processed foods could compete directly with seeds for ant attention, diverting foragers away from diaspores and reducing seed removal. Alternatively, food subsidies could intensify ant recruitment and activity, indirectly increasing seed encounters. Evidence from urban systems suggests that ants exposed to anthropogenic resources may modify their foraging behavior and resource preferences, reallocating effort toward alternative food sources and thereby potentially altering the strength and nature of mutualistic interactions [10,11,12]. Yet, whether such effects extend beyond urban habitats into less disturbed forests, and whether they persist across distinct ant assemblages, remains largely unexplored.

Most existing studies on food subsidies and ecological interactions focus on vertebrates [13], leaving a critical gap in our understanding of how human food waste affects invertebrate-mediated ecosystem processes. In tropical systems, where ants are dominant seed removers and secondary dispersers [4,14], even subtle changes in ant behavior could have disproportionate effects on seed fate and plant regeneration. Moreover, differences in ant species composition between urban and forest habitats [15,16] raise the possibility that functional redundancy (i.e., multiple species performing similar ecological roles, such as seed removal or dispersal) or the presence of seed-specialist ants might buffer the impacts of anthropogenic food inputs in more diverse communities.

Here, we experimentally test whether common human food residues disrupt ant-mediated seed removal, ant activity, and ant species composition in two contrasting habitat contexts (urban and forest) in Panama. Using potato chips and cookies as representative processed foods, which are common forms of anthropogenic food waste and are readily exploited by ants due to their high lipid and carbohydrate content [9], we conducted a series of field experiments to (1) quantify how food waste affects seed removal rates, (2) assess whether these effects are consistent across habitats with distinct ant assemblages, and (3) disentangle behavioral distraction from physical obstruction by manipulating the spatial arrangement of food items. By integrating survival analyses, ant activity data, distance gradients, and community composition analyses, our study provides a mechanistic assessment of how anthropogenic food subsidies interfere with a key ecological interaction at fine spatial scales.

## 2. Materials and Methods

We conducted three consecutive field experiments to test whether food waste items (chips or cookies) influence seed removal by ants. In all experiments, we used oat seeds (*Avena sativa*) placed in open plastic lids from 90-mm Petri dishes (seed depots), which were inverted and gently pressed into the soil so that their edges were flush with the ground surface. This seed type was chosen based on prior work showing its consistent removal by ants in tropical field experiments [17], making it a suitable standardized model for testing interference effects. Although *Avena sativa* is not native to the study system, and lacks specialized traits such as elaiosomes, it is readily collected by opportunistic and granivorous ants and has been shown to be among the most frequently removed seed types in comparable studies [18]. Its use allows for controlled comparisons of ant foraging responses across treatments without confounding variation in seed traits such as size, nutritional content, or chemical defenses.

We arranged seed depots in a 2 × 9 grid, with 9 m spacing between depots and alternating depot treatments. Grids monitored by different author pairs were separated by at least 100 m to ensure spatial independence among experimental units. Grids were established in relatively homogeneous areas within each site to minimize variation in environmental conditions such as vegetation cover and ground substrate. In all sites, seed depots were placed under tree cover to avoid direct exposure to sunlight and reduce temperature variability among depots. Trials ran from approximately 9:00 AM to 12:00 PM (120 min), during which teams of student pairs (16 authors total) monitored depots every 5 min. This time window was selected to capture diurnal foraging activity, when many ground-foraging ants are active, and to standardize comparisons across treatments and sites [19]. While some ant species may be primarily crepuscular or nocturnal, our objective was to quantify how anthropogenic food residues affect daytime seed removal dynamics, which represent a major component of ant-mediated seed fate. At each time point, we recorded both seed removal and the number of ants present on the lid. Only ants physically present on the surface of the lid were counted to standardize observations. Seeds were considered removed only when they were no longer present on the lid, ensuring a consistent and unambiguous criterion across observers. All observers were instructed using a standardized protocol prior to data collection, including clear definitions of ant counts and seed removal, to minimize observer-related variability and conducted brief training and calibration sessions before the experiments. In the second experiment, we collected individuals observed on the depots for later taxonomic identification.

In all experiments, we used Lay’s Original^®^ potato chips (Frito-Lay Inc, Orlando, FL, USA) and, for the second experiment, we included the chocolate cookie portion of Oreo^®^ cookies (i.e., without the white cream filling; Mondelēz International, New York, NY, USA). We used comparable amounts of chips and cookie fragments, matching weights as closely as possible (~2.6 g) to standardize the quantity of food provided across treatments. Although chips and cookies differ in nutritional composition (e.g., fat and carbohydrate content), our objective was not to compare specific nutritional effects but to test whether common processed food residues influence ant foraging behavior. Both food types are representative of widely available anthropogenic food waste and are readily exploited by ants in urban environments [9].

### 2.1. First Experiment (Pilot Test: Seed Removal)

The first experiment was conducted at the urban site on the campus of the University of Panama (UP, N 8° 59′ 1.47″, W 79° 31′ 58.9″). Here, we tested whether chips influenced seed removal when placed in a ring around the entire perimeter of the seed depot, at a distance of approximately 30 cm from the center. The ring was intended to be continuous, forming a closed circle of food material around the depot, although small unintentional gaps may have occurred. This design was used to maximize the potential for both behavioral distraction and physical obstruction. The 30 cm distance was selected as a standardized intermediate distance that allowed clear separation between the seed depot and the food items while maintaining close proximity likely to influence ant foraging decisions. Control depots contained seeds only. This experiment included 2520 seeds distributed across 63 depots per treatment. This pilot revealed that seeds surrounded by chips were removed more slowly than control seeds (see Section 3), suggesting that the presence of chips reduced ant activity. Field observations confirmed that ants interacted with the chips, often transporting them, which may have distracted them from the seeds. However, because chips formed a continuous ring around the seeds, this design could not distinguish whether reduced seed removal resulted from behavioral distraction or from a physical barrier effect, motivating the more conservative designs used in subsequent experiments.

### 2.2. Second Experiment (Seed Removal and Ant Activity)

To address the possibility that the effect observed in the first experiment was due to a physical barrier, we conducted a second experiment using a more conservative design. In this setup, food items were placed only on one side of the seed depot rather than forming a complete ring, thereby reducing, but not completely eliminating, potential spatial obstruction, as food placement on one side may still introduce asymmetry in ant access to the depot. The experiment was conducted at both an urban site (University of Panama, UP) and a forested site (Pipeline Road at Soberanía National Park, PLR, 9° 7′ 57.1″, W 79° 43′ 6.6″), using three treatments: (1) control (seeds only), (2) chips (seeds + crumbled potato chips on one side), and (3) cookies (seeds + crumbled cookie pieces on one side). We used the black cookie shell of Oreo cookies (white filling removed) and Lay’s Original flavor potato chips. The side on which food items were placed was alternated across depots and replicates to control for potential directional biases. Both items were manually crumbled into small fragments by hand to simulate residues generated during human consumption, where chips and cookies are often broken into smaller pieces rather than discarded intact. Fragmentation also facilitated access and handling by ants. Although fragment size was not measured precisely, we maintained consistency by preparing all food items in a similar manner and standardizing the total mass provided per depot (~2.6 g). To avoid contamination and eliminate human odor cues, all food handling was performed using latex gloves. This experiment included 5756 seeds distributed in 96 depots for chips, 100 depots for control and 94 depots for cookies. Minor differences in the number of depots among treatments resulted from logistical constraints during field deployment. Treatments were interspersed within grids, and all analyses were conducted using statistical models that are robust to unbalanced sample sizes.

This design allowed us to test: (1) whether different types of food waste altered ant-mediated seed removal and ant activity (measured as the number of ants present on the seed depot at each observation interval), (2) whether such effects were consistent across contrasting habitats, and (3) whether the patterns observed in the first experiment could be explained by more than just spatial obstruction.

### 2.3. Third Experiment (Distance Gradient)

To further explore the mechanisms behind the reduced seed removal observed in the presence of food waste, we conducted a third experiment in which potato chips were placed at increasing distances from the seed depot. This experiment tested whether the proximity of chips influenced ant foraging behavior and seed removal. We used a design similar to previous experiments, with control depots (seeds only) and treatments where chips were placed at 0 cm, 30 cm, or 60 cm from the center of the seed lid on one side of the depot. The selected distances represent a gradient of proximity between seeds and food items, ranging from direct contact (0 cm) to increasing spatial separation, allowing us to assess how the influence of food residues on ant foraging behavior changes with distance. Seed removal analyses were based on 1648 seeds distributed across 12 depots per treatment, monitored every 5 min for 120 min, and ant presence was recorded. All observations in this experiment were conducted by a single observer (BM), eliminating inter-observer variability. Ant activity was quantified as the number of ants present on the surface of the seed depot, and seed removal was recorded only when seeds were no longer present on the depot surface.

### 2.4. Statistical Analysis

We analyzed seed removal over time using Cox proportional hazards models, with seed survival (i.e., not removed) as the event of interest. Time-to-removal data were structured as right-censored survival objects (Surv), with status coded as 1 for removed seeds and 0 otherwise. For experiment 1, the model included treatment (control and chips). For experiment 2, the model included treatment (control, chips, cookies), site (PLR, UP), and their interaction as fixed effects. To account for non-independence of depots sampled repeatedly in all experiments, we included clustered standard errors by depot ID. We used the coxph() function from the survival package and computed estimated marginal means (EMMs) and pairwise hazard ratios via the emmeans package (type = “response”). Results were back-transformed to obtain hazard ratios and percent changes in seed removal relative to the control. We visualized survival curves using the survminer package, including 95% confidence intervals.

Poisson models for ant visitation data showed evidence of overdispersion in experiment 2; therefore, we fitted negative binomial generalized linear mixed models (GLMMs) to analyze the total number of ants observed on seed depots. Fixed effects included site (PLR, UP), treatment (control, chips, cookies), and their interaction. The model included random intercepts for couple ID (observer pair) and nested depot ID, to account for repeated measures across sampling units and potential variability among observer teams. Models were fitted using the glmer.nb() function from the lme4 package with Laplace approximation. Estimated marginal means and pairwise contrasts were obtained using the emmeans package and back-transformed to the response scale to quantify proportional and percentage reductions in ant activity relative to the control.

To test whether ant species composition differed between the forest (PLR) and urban (UP) sites in experiment 2, we performed a multivariate community analysis based on ant visitation data. We constructed a site-by-species matrix using all ant species columns in the dataset, with rows corresponding to individual observation intervals. Community dissimilarity among samples was quantified using Bray–Curtis distances, calculated with the vegdist() function in the vegan package. Differences in ant species composition between sites were tested using PERMANOVA (permutational multivariate analysis of variance) implemented with the adonis2() function, with site (UP vs. PLR) as the explanatory factor and 999 permutations. To verify that significant PERMANOVA results reflected differences in community composition rather than differences in multivariate dispersion, we tested for homogeneity of dispersion between sites using PERMDISP, implemented via the betadisper() function followed by an ANOVA on distances to group centroids. All community analyses were conducted in R (v 4.5.3) using the vegan package.

## 3. Results

### 3.1. First Experiment (Pilot Test: Seed Removal)

**Figure 1:**
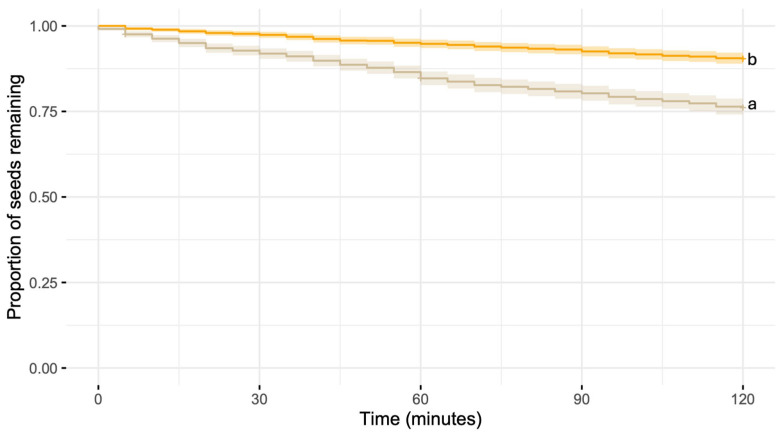
Kaplan–Meier survival curves showing oat seed removal by ants in an urban site. Seeds were left untreated (control; cream line) or surrounded by potato chips (orange line). Curves represent the proportion of seeds remaining over time. Shaded areas around each curve indicate 95% confidence intervals. Different letters depict significant differences.

Seed removal was significantly reduced when potato chips fully surrounded the seed depots. Seeds in control depots were removed 2.72 times faster than seeds surrounded by chips (hazard ratio [HR] = 2.72, 95% CI: 1.66–4.45; z = 3.99, *p* < 0.001, Figure 1).

### 3.2. Second Experiment (Seed Removal and Ant Activity)

**Figure 2:**
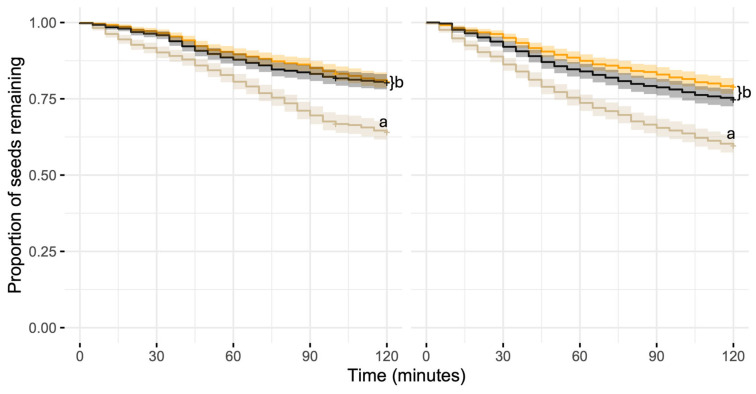
Kaplan–Meier survival curves showing oat seed removal by ants in a forest site (**left panel**) and an urban site (**right panel**) in Panama. Seed depots were either left untreated (control; cream line) or exposed to nearby food residues consisting of cookie pieces (black line) or potato chips (orange line). Curves represent the proportion of seeds remaining over time, and shaded areas indicate 95% confidence intervals. Different letters denote significant differences among treatments.

Seed removal was significantly affected by treatment in both habitats. Pairwise contrasts using estimated marginal means (EMMs) revealed that seeds in the control treatment were removed significantly faster than those near either food item (chips or cookies) in both forest (PLR) and urban (UP) sites. At the urban site, control seeds were removed 2.21 times faster than seeds near chips (HR = 2.21, *p* = 0.001, Figure 2) and 1.78 times faster than seeds near cookies (HR = 1.78, *p* = 0.03, Figure 1). In the forest, control seeds were removed 2.07 times faster than seeds near chips (HR = 2.07, *p* = 0.05, Figure 2) and 1.99 times faster than seeds near cookies (HR = 2.00, *p* = 0.04). Chips and cookies did not differ significantly from each other in either site (urban: HR = 0.80, *p* = 0.68; forest: HR = 0.96, *p* = 0.99, Figure 2), indicating that both types of food disrupt seed removal similarly regardless of ant community composition (Figure 2).

**Figure 3:**
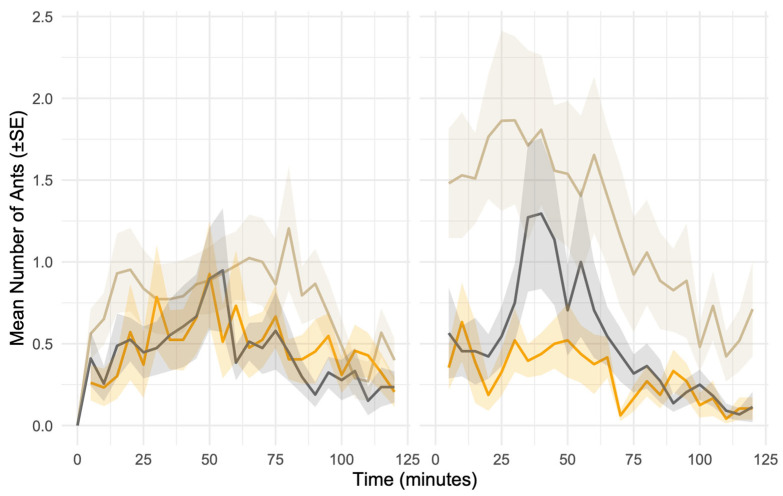
Ant activity over time during seed removal experiments in a forest site (**left panel**) and an urban site (**right panel**) in Panama. Ants were counted every 5 min for 120 min at seed depots under three treatments: control (cream line), cookies (black line), and chips (orange line). Lines represent mean ant abundance per depot through time, and shaded bands indicate ± SE.

Ant activity near seeds was significantly reduced in the presence of chips (Estimate = −0.626, z = −2.93, *p* = 0.003) and cookies (Estimate = −0.690, z = −3.51, *p* < 0.001), relative to the control treatment (Figure 3). The effect of chips varied strongly between sites, as shown by a significant interaction with site (Estimate = −0.915, z = −2.80, *p* = 0.005), indicating a greater suppressive effect in the urban site (UP) than in the forest. The interaction between site and cookies was not significant (Estimate = −0.426, *p* = 0.18), suggesting a more uniform effect of cookies across both habitats. Notably, baseline ant activity under the control treatment was significantly higher in the urban site than in the forest (Estimate = 0.833, z = 3.32, *p* < 0.001, Figure 3), highlighting that while urban ants were more active overall, they were also more sensitive to food distractions.

Ant species composition differed significantly between sites, with 9.4% of the variation in community structure explained by habitat type (PERMANOVA: R^2^ = 0.094, F = 159.18, *p* = 0.001). This confirms that the observed differences in seed removal and ant activity are accompanied by shifts in the composition of foraging ant assemblages across the forest and urban environments. A test for homogeneity of multivariate dispersion (PERMDISP) showed no significant differences in within-group variance between sites (F = 0.88, *p* = 0.35), indicating that PERMANOVA results reflect true compositional differences rather than differences in dispersion. A total of 9 ant species were recorded at the urban site (UP) and 8 at the forest site (PLR) (Figure S1), with 3 species shared between sites.

### 3.3. Third Experiment (Distance Gradient)

**Figure 4:**
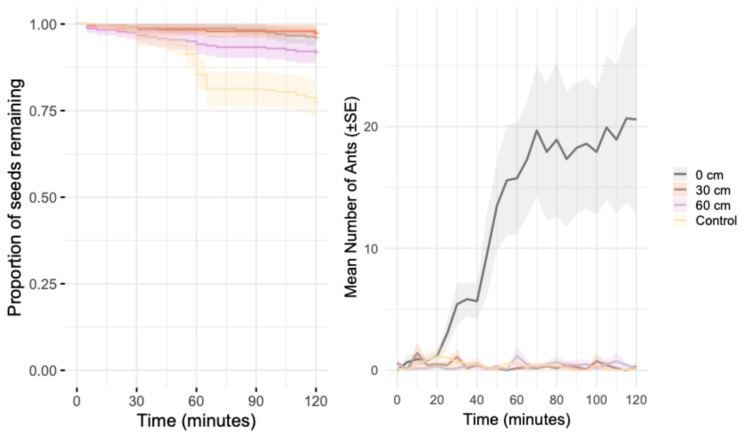
Distance-gradient effects of chips on seed removal and ant activity. (**Left panel**) Kaplan–Meier survival curves showing the proportion of seeds remaining through time (0–120 min) under a chip treatment placed at different distances from the seed depot (0, 30, and 60 cm) and a control without chips. Shaded bands indicate 95% confidence intervals. (**Right panel**) ant activity through time for the same treatments, with ants counted at 5-min intervals; lines represent the mean number of ants per depot and shaded bands indicate ± SE. Distances indicate the separation (cm) between the chips and the seed depot.

Seeds in control treatments were removed faster than chips placed at 0 cm (z = −2.0, *p* = 0.04, Figure 4) and at 30 cm (z = −2.35, *p* = 0.02, Figure 4) but there was no difference in seed removal when chips were placed at 60 cm as compared to the control (z = −1.43, *p* = 0.16, Figure 4). No significant differences in seed removal were detected among chip treatments placed at 0, 30, or 60 cm from the seed depot (Table S1, Figure 4).

The highest activity of ants on the depots was at 0 cm as compared to the control (Estimate = 3.42, t = 5.8, *p* = 0.0001, Figure 4), with no differences at 30 cm (Estimate = −0.17, t = 0.61, *p* = 0.79, Figure 4) and 60 cm (Estimate = −0.11, t = 0.61, *p* = 0.85, Figure 4) as compared to the control.

The rate of seed removal was strongly influenced by the number of ants observed, but this effect varied across treatments. In the absence of chips (control), the presence of ants significantly increased the likelihood of seed removal over time (hazard ratio [HR] = 2.71, z = 5.79, *p* < 0.001). However, this positive effect of ant abundance was significantly reduced in chip treatments, particularly at 0 cm (interaction: HR = 0.36, z = −5.93, *p* < 0.001) and 30 cm (HR = 0.37, z = −2.61 *p* = 0.009), while the reduction at 60 cm was weaker and not statistically significant (HR = 0.55, z = −1.07, *p* = 0.28). 

## 4. Discussion

Our results demonstrate that common anthropogenic food residues—namely chips and cookies—disrupt ant-mediated seed removal across two ecologically distinct environments: an urban site and a relatively intact forest. Across all experiments, seeds placed near human food items were removed significantly more slowly than control seeds, indicating a clear disruption of ant–seed interactions. Importantly, this disruption occurred despite differences in ant species composition between the two habitats, highlighting a generalizable functional impact that is not contingent on particular species identities [9,18,20].

All interpretations presented below are based on experimentally observed changes in seed removal rates, ant activity, and their spatial relationships with food residues. This is one of the few empirical studies to evaluate how food subsidies from human sources impact invertebrate mutualisms, a dynamic more commonly explored in vertebrate ecology [13]. By combining seed removal rates, ant activity patterns, and distance experiments, our findings extend previous observations on urban ant diets and their incorporation of processed food items [8,21] and reveal a mechanistic pathway (behavioral distraction and spatial aggregation) through which food waste impairs ecosystem functions.

Such impairment was generated by both chips and cookies by significantly reducing seed removal rates in forest (PLR) and urban (UP) sites. In both habitats, control seeds were removed approximately twice faster than those near chips. Similar reductions were observed for cookies, and no significant differences emerged between chips and cookies, suggesting convergent effects of these food types. This finding underscores that even in forest sites with low prior exposure to processed foods, and despite the potential buffering capacity of higher ant diversity [15,16], which might include more specialized seed-foraging species, food residues can override normal foraging behavior. The assemblages at both sites were dominated by generalist foragers rather than specialist seed-harvesting ants, which likely contributed to the similar magnitude of treatment effects across habitats. This suggests that the disruptive effect of food residues is not restricted to systems dominated by specialist granivores, although future studies in communities with a higher proportion of seed specialists may reveal different outcomes.

While forest ants may rely more on natural seed resources, urban ant communities—accustomed to frequent food waste—may experience more intense distraction, potentially driven by shifts in foraging strategies, past exposure, or nutritional preferences [15,22]. These behavioral shifts were reflected in ant activity: ant foraging for seeds decreased significantly in the presence of food items, with the strongest suppression observed in the urban site for the chip treatment. Although urban ants were more active overall, their heightened sensitivity to food residues suggests that resource-rich but ephemeral environments may promote rapid and localized aggregation [21,23], potentially amplifying interference with native food sources.

Our distance experiment showed that the disruptive effect of chips on seed removal is both strong and spatially localized. Even when seeds were placed directly adjacent to chips (0 cm), ants did not prioritize them, indicating that the presence of human food residues can override typical foraging behavior. This is particularly striking because seed removal typically increases with proximity and visibility [24,25]. Although *Avena sativa* is not native and lacks elaiosomes, its consistent removal by ants makes it a suitable model for general foraging responses. The pattern of disruption observed here is consistent with other systems where well-established ant mutualisms are overridden by anthropogenic inputs. For example, Boulay et al. [26] showed that the presence of artificial sugar resources can alter ant foraging priorities, reducing investment in other mutualistic interactions. Similarly, in ant–hemipteran systems, the availability of alternative sugar sources can decrease tending behavior and modify interaction strength [27]. Another local example occurs in the defensive mutualism between *Pseudomyrmex spinicola* ants and *Vachellia collinsii* plants. Field observations show that sugar cane solutions can distract ants from collecting extrafloral nectar, possibly reducing plant protection and facilitating nectar exploitation by opportunistic visitors such as dipterans (D.G., pers. obs.). These patterns indicate that highly rewarding anthropogenic resources can redirect ant foraging effort away from natural partners, thereby altering the outcome of mutualistic interactions. This suggests that the effects we document are likely to extend beyond the specific seed type used.

This supports the view that behavioral distraction and not just species loss can degrade ecological interactions [28]. In our case, under control conditions, seed removal increased with ant abundance, as expected for functionally mutualistic interactions. However, in the presence of alternative high-reward food items (chips or cookies), this positive relationship was disrupted: even when ants were present near depots (0 cm), they were less likely to remove seeds. This pattern aligns with previous work showing that alternative food sources can distract ants from tasks like granivory or prey retrieval [9,26], highlighting behavioral interference as a critical, and often overlooked, mechanism in mutualism disruption. The spatial attenuation of this effect (diminishing by 60 cm) highlights the fine-scale nature of these disruptions. Localized food waste patches can generate microzones of functional decay in seed dispersal services, potentially reshaping seed fate and plant recruitment patterns across heterogeneous landscapes [26]. Recent syntheses support this view. A global meta-analysis showed that anthropogenic disturbance consistently reduces ant-mediated seed dispersal across biomes, even where ant communities persist, indicating that human impacts often homogenize ecological interactions through behavioral disruption rather than simply species loss [29].

As part of the plant recruitment process, ants act as both seed dispersers and predators, and reductions in removal rates may increase seed accumulation near parent plants, elevating risks from density-dependent mortality due to predation or pathogens [30,31]. The consistent reduction in seed removal across treatments and sites suggests that human food residues may interfere with early stages of plant regeneration, even in relatively undisturbed forests. Because our study included only one urban and one forest site, it was not designed as a replicated comparison of habitat types, and differences between UP and PLR should be interpreted cautiously. Instead, our goal was to test whether the disruptive effect of anthropogenic food residues on ant-mediated seed removal occurs across distinct habitat contexts. The consistent response observed in both sites, despite differences in species composition, suggests that this effect is not restricted to a particular local assemblage and may reflect a general behavioral response to food subsidies [8,9]. These findings further suggest that, in our study system, anthropogenic food residues can alter ant foraging behavior in ways that may contribute to the homogenization of ant–seed interactions across distinct local assemblages, consistent with evidence that urbanization not only alters species composition [9,15,22] but also influences ecological interactions through behavioral plasticity (e.g., [11]) and subsidy-driven foraging.

Further work is needed to understand whether an increase in the proportion of true seed-specialist ants, especially in forest habitats, could buffer the negative effects of human food inputs. In our study, some species like *Paratrachymyrmex cornetzi* foraged primarily on seeds, often ignoring chips and cookies, suggesting trait-based resilience. Likewise, Boulay et al. [26] reported that *Aphaenogaster iberica* remained effective seed dispersers despite the presence of sugary distractions. These findings suggest that functional traits, rather than species identity alone, may determine mutualism robustness under anthropogenic pressures. In that line, other studies should include long-term exposure experiments and true myrmecochorous seed species to assess whether certain ant groups (e.g., Attini) maintain their ecological roles under sustained food subsidy conditions. Moreover, understanding how food waste interacts with habitat structure, predator presence, and thermal constraints (e.g., shifts to nocturnal foraging under heat stress; [32]) will help identify context-dependent vulnerabilities in ant–seed mutualisms.

Although our results are restricted to diurnal foraging periods, the consistent effects observed across sites and treatments suggest that anthropogenic food residues can disrupt seed removal under common daytime conditions. Whether similar patterns occur during nocturnal or crepuscular activity remains an open question. Ultimately, our study highlights that even localized food waste can restructure ant foraging behavior and disrupt key ecosystem functions. When considered alongside experimental, regional, and global evidence [29,33], our results suggest that anthropogenic food subsidies contribute to the functional homogenization of ant–seed interactions across distinct local assemblages. If similar processes occur in other systems, these effects could become more pronounced as urbanization expands, underscoring the need to consider indirect, behavioral mechanisms in conservation strategies aimed at preserving ecological interactions.

## 5. Conclusions

Our study demonstrates that common human food leftovers can substantially disrupt ant-mediated seed removal in both forest and urban habitats. Across three experiments, seeds placed near cookies or potato chips were consistently removed more slowly than control seeds, despite differences in ant assemblages between habitats. The suppression of seed removal was strongest at close proximity, indicating that the effect operates at a fine spatial scale. Moreover, the positive relationship between ant abundance and seed removal observed under control conditions weakened or disappeared in the presence of food residues, supporting the interpretation that behavioral distraction rather than simple physical obstruction underlies the observed patterns.

These findings suggest that anthropogenic food subsidies can interfere with ecologically important ant–plant interactions even in relatively intact tropical forests. Because ants influence seed fate, plant recruitment, and ultimately forest structure, subtle shifts in foraging decisions may scale up to affect community dynamics over time. The presence of processed food waste may therefore represent an underappreciated pathway through which human activities alter ecosystem functioning.

Reducing food litter in both urban and natural areas could help preserve natural foraging behavior and maintain the ecological services provided by ants. Understanding how human subsidies reshape species interactions is essential for predicting the broader consequences of global change on tropical ecosystems.

## Data Availability

The datasets generated for this study can be found as Supplementary Material (Table S2).

## Supplementary Materials

The following supporting information can be downloaded at: https://www.mdpi.com/article/10.3390/biology15080657/s1. Table S1: Pairwise comparison of survival analysis on seed removal of batches of seeds in Experiment 3; Table S2: Raw data; Figure S1: Ant species composition per site.
