# Supplementary material for: Cookies, Chips, and Seeds: How Human Food Leftovers Influence Ant-Mediated Seed Removal
Source: Biology (Basel). 2026 Apr 21;15(8):657. doi: 10.3390/biology15080657 (PMC13113908; doi:10.3390/biology15080657)
Supplement: Supplementary file 1 [file biology-15-00657-s001.zip › Supplementary_Material.pdf]

Supporting information for manuscript Cookies, chips, and seeds: how human food leftovers influence ant-mediated seed removal.

Table S1. Pairwise comparison of survival analysis on seed removal of batches of seeds in Experiment 3, placed with chips at different distances (0, 30 and 60 cm) or without chips (control).

comparison	estimate	Z ratio	p
Control – 0 cm	1.89	2.01	0.04
Control – 30 cm	2.29	2.35	0.02
Control – 60 cm	1.11	1.4	0.15
0 cm – 30 cm	0.40	0.36	0.72
0 cm – 60 cm	-0.783	-0.83	0.41
30 cm – 60 cm	-1.19	-1.2	0.23

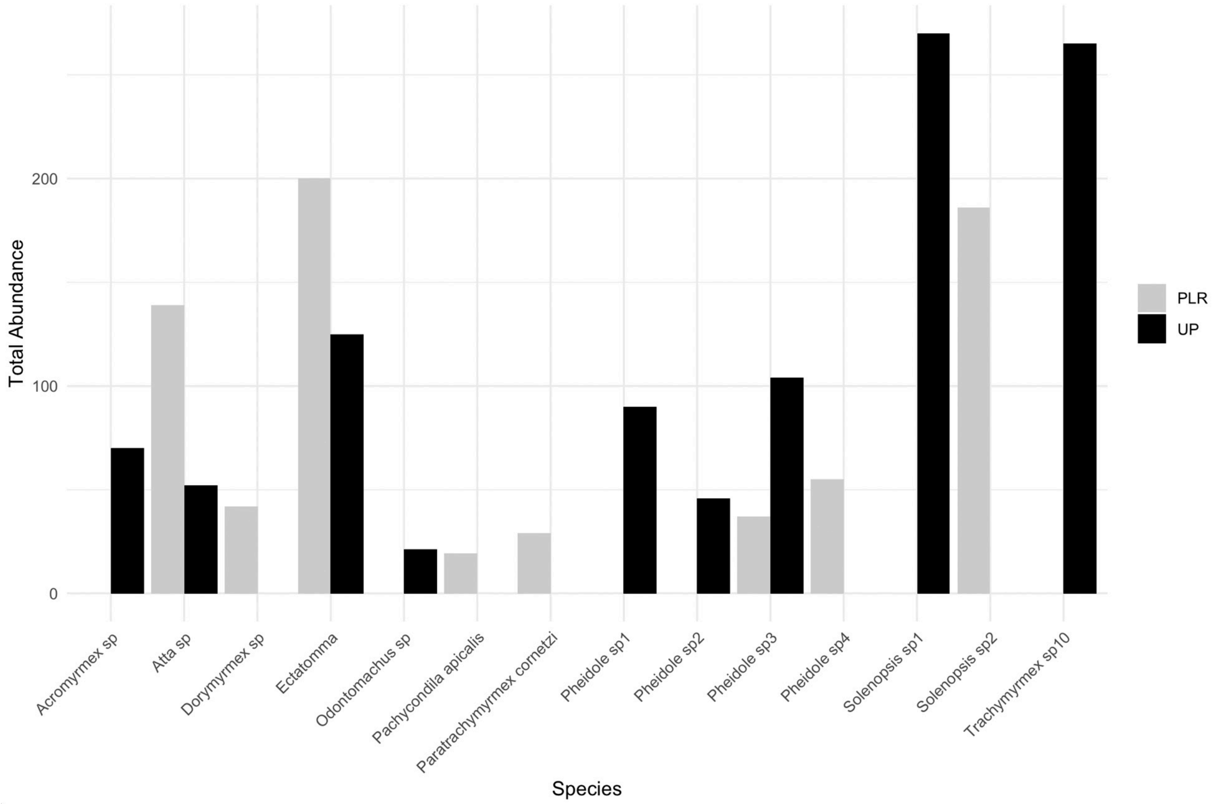

Figure S1. Ant species composition and total abundance at seed depots in the urban (UP) and forest (PLR) sites. Bars represent the cumulative number of individuals recorded per species across all observation intervals during Experiment 2. Colors indicate site (black = UP; gray = PLR).
